# Safety Concerns in Neurological Clinical Trials: A Challenge That the FDA Must Resolve

**DOI:** 10.3390/biomedicines12122918

**Published:** 2024-12-22

**Authors:** Sarfaraz K. Niazi

**Affiliations:** College of Pharmacy, University of Illinois, Chicago, IL 60612, USA; sniazi3@uic.edu; Tel.: +312-297-0000

**Keywords:** Alzheimer’s disease, FDA, lecanemab, donanemab, aducanumab, NY Times, clinical trials, brain bioavailability, human abuse

## Abstract

**Background:** Monoclonal antibodies approved by the FDA, lecanemab, donanemab, and aducanumab, are failing to meet the expected efficacy to treat early Alzheimer’s disease, and aducanumab has been recalled. **Methods**: Recently, it was reported that the clinical trials of these antibodies may have violated patient’s rights and subjected them to high, likely lethal risk. The challenge with developing antibodies to treat neurological disorders is their poor blood–brain barrier (BBB) penetration if the antibody must enter the brain, resulting in almost negligible brain bioavailability, requiring high dosing that can be toxic. **Results**: The reported efficacy of these drugs should also be reviewed, considering the placebo effects, since all antibodies have shown severe side effects that are not prevented by the placebo responses. In this critical and urgent advice to the FDA, I am suggesting a guideline amendment to all clinical trials requiring proof of sufficient brain bioavailability at the site of action, where it is known. **Conclusions**: For antibodies to cross the blood–brain barrier, there are proven options such as conjugating with transferrin protein, making clinical trials in its absence more questionable.

## 1. Introduction

On 23 October 2024, the New York Times front page reported its finding “What Drugmakers Did Not Tell Volunteers in Alzheimer’s Trials”, which disclosed serious concerns about the clinical trials conducted for Alzheimer’s drugs like Eisai’s Leqembi (lecanemab-irmb) and Eli Lilly’s Kisunla (donanemab-azbt) [1]. It describes how drug companies failed to disclose critical genetic test results to participants, especially regarding the APOE4 gene variant, which increases the risk of brain injuries in patients receiving these drugs [2]. By 2021, about 2000 participants had enrolled in the Leqembi trials, among them a high-risk subset predisposed to brain bleeding and swelling [3,4]. Since the penetration across the blood–brain barrier (BBB) is limited, the observed effects likely required very high, likely toxic doses to demonstrate clinical responses.

Despite informing volunteers about potential genetic testing, companies withheld individual results, a decision now seen as a violation of informed consent principles by bioethicists. Two high-risk volunteers died, and over 100 experienced brain bleeding or swelling, some with severe consequences. Eisai’s Leqembi received FDA approval in 2023 under an accelerated approval process despite its safety concerns, which should not have shown only a modest cognitive benefit [5]. In 2024, the FDA called a meeting to discuss whether it should receive traditional approval. Despite many serious concerns, including those of the author, the FDA granted traditional approval [3]. However, regulatory agencies in the European Union [6] first rejected and then accepted and in Australia [7] rejected it due to its temporary and limited efficacy compared to its risks.

## 2. Testing Antibodies to Treat Neurological Disorders

Lecanemab selectively binds to soluble protofibrillar forms of amyloid-beta, effectively reducing amyloid plaques, as confirmed by amyloid PET scans. The Phase 3 CLARITY AD trial showed that lecanemab slowed cognitive decline by 27% compared to placebo in early Alzheimer’s patients, significantly improving cognitive and functional outcomes [8]. It is important to understand that the placebo effects are more common with trials involving neurological responses; however, there is little consistency in the control group, as well as the treatment group, making it questionable if a trial judged based on comparison with placebo data can represent the real-world use of the drug.

Similarly, donanemab targets a specific epitope on amyloid-beta plaques and has demonstrated rapid amyloid clearance, with many patients achieving amyloid-negative status. In the TRAILBLAZER-ALZ 2 trial, donanemab slowed cognitive decline by 35% in patients with low to intermediate tau levels, suggesting its effectiveness in early disease stages [9]. The primary and secondary clinical outcome measures for Lecanemab, donanemab, and aducanumab were all very small in relation to the range of the outcome tests used. Therefore, although the reported changes were statistically significant, their clinical significance is very low.

Aducanumab, which binds aggregated amyloid-beta, also demonstrated substantial amyloid reduction, but its clinical efficacy was inconsistent. While the EMERGE trial reported a modest slowing of cognitive decline at high doses, the ENGAGE trial failed to show statistically significant benefits, raising concerns about its clinical impact despite amyloid clearance [10]. These results highlight the potential of amyloid-lowering therapies to modify disease progression but underscore the need for further research to confirm the relationship between amyloid reduction and cognitive benefits. 

The risks associated with amyloid-beta, tau protein, and alpha-synuclein-targeting drugs extend beyond immediate brain injuries, with studies suggesting a higher mortality rate among those treated with antibodies than untreated Alzheimer’s patients. There is also emerging evidence that these drugs may accelerate brain shrinkage, further compounding concerns about their long-term safety. Considering these findings, the clinical trials exemplify the ethical and medical complexities of advancing treatments for neurodegenerative diseases [11].

The relationship between amyloid-beta, tau protein, and alpha-synuclein-targeting drugs and mortality rates among treated patients is an emerging area of research, particularly in neurodegenerative diseases like Alzheimer’s disease and Parkinson’s disease.

Three antibodies targeting amyloid-beta (donanemab, aducanumab, lecanemab) have been approved, but none that are anti-tau and anti-alpha-synuclein, though many of these, along with amyloid-beta targeting antibodies, are under investigation. Some studies have reported an increase in mortality or serious adverse events in patients treated with these antibodies compared to controls, particularly in those with more advanced disease or certain comorbidities [12]. Similar observations were reported with treatments targeting tau protein [8] and the alpha-synuclein-targeting antibodies (e.g., prasinezumab) [13]. Many of the concerns about mortality have emerged from post hoc analyses of clinical trial data, emphasizing the need for ongoing monitoring and long-term studies. This concern is now further highlighted in the recent NY Times investigation.

While companies continue to pursue the development of these drugs, scientists suggest a broader focus on alternative therapeutic avenues, such as reducing inflammation or enhancing blood flow, as the amyloid theory alone appears insufficient to address neurological disorders’ multifaceted nature. Table 1 lists the antibodies for which clinical trials were conducted and failed or withdrawn after approval for lack of efficacy or high toxicity (Table 1) [14].

Currently, there are 30 studies listed in clinical trials to treat Alzheimer’s disease (https://clinicaltrials.gov/search?cond=Alzheimer%27s%20Disease&term=Monoclonal%20Antibody; accessed on 25 October 2024), and none for Parkinson’s disease. Table 2 lists the approved monoclonal antibodies (Table 2).

## 3. The Future

While awareness of the flaws in clinical trials of neurological disorder treatments with antibodies has begun to rise, a more significant issue, whether these drugs even qualify to be tested in the first place based on their lack of bioavailability in the brain, has been widely ignored [24]. The known brain bioavailability of these antibodies is 0.01–0.1% [25], requiring high dosing to capture the minute effects; this shows that these antibodies are highly effective, but their inevitable high dosing makes them a significant clinical concern because of the side effects responsible for the toxicity of these antibodies. There is also a possibility that these effects are placebo-driven, as commonly found in such treatments [26,27,28,29]. Notably, placebo effects are primarily found in clinical trials where neurological symptoms are recorded as proof of efficacy.

The issue of ensuring brain bioavailability becomes more relevant since there are known mechanisms to improve the entry of antibodies into the brain. Antibodies can be readily made effective and safe by engineering them to go through transcytosis [30] such as by binding the antibodies with transferrin protein or its N-methyl lobe using a cleavable linker to avoid exocytosis, increasing the half-life of these antibodies in the brain [31].

Binding antibodies with transferrin protein or N-methyl lobe with a cleavable linker is the most optimal engineering method. To demonstrate that such binding does not adversely impact the efficacy of these antibodies, we employed bioinformatic modeling to investigate the impact of non-cleavable linkers (G4S)n in antibody binding.

Our examination, centered on steric hindrance, sought to understand the effects of the length of the linker and its influence on the binding avidity with the Aβ protein.

In addition to the clearance pathways, the behavior of antibodies in the brain can vary depending on their affinity for specific target antigens. For instance, antibodies designed to bind high-affinity receptors, such as those on transport mechanisms like the transferrin receptor or therapeutic targets such as amyloid-beta in Alzheimer’s disease, may experience enhanced retention due to these interactions [32].

**Figure 1:**
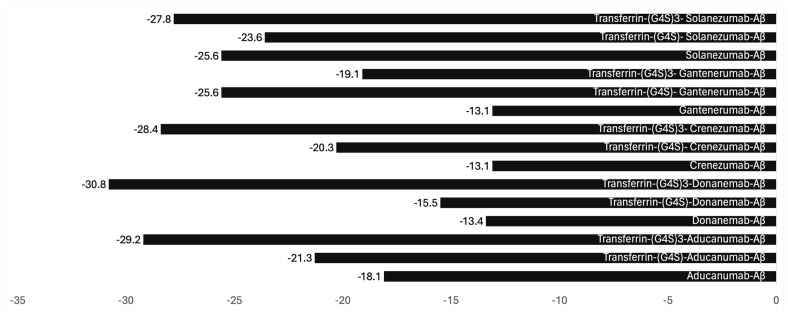
Binding properties ΔG (kcal mol^−1^) of antibodies with amyloid-beta with transferrin conjugation and small and long linkers (created using https://www.cgl.ucsf.edu/chimera/; https://alphafoldserver.com/about; and https://rascar.science.uu.nl/haddock2.4/, accessed on 25 October 2024). The Gibbs free energy change (ΔG\Delta GΔG) in binding studies is closely related to the interactions between Interacting Components (ICs) and Non-Interacting Species (NIS), playing a critical role in determining the strength and stability of molecular interactions. Interacting Components (ICs) are species such as molecules or ions that engage in specific interactions like binding, forming complexes, or undergoing reactions. Electrostatic interactions, hydrogen bonds, van der Waals forces, and hydrophobic effects drive these interactions. When ICs bind to each other, they form a complex, and the ΔG\Delta GΔG of this binding process reflects the stability of the complex. A negative ΔG\Delta GΔG indicates a spontaneous and favorable interaction, meaning that the ICs have a strong affinity for each other and form a stable complex. The more negative the ΔG\Delta GΔG, the stronger the interaction between the ICs, such as in the case of charged–charged ICs, which often exhibit powerful interactions due to electrostatic forces, leading to a more negative ΔG\Delta GΔG. Charged–polar ICs, which exhibit a combination of electrostatic and dipole interactions, generally result in moderately negative ΔG\Delta GΔG. In contrast, polar–polar ICs, driven by hydrogen bonding and dipole–dipole interactions, also contribute to a moderately negative ΔG\Delta GΔG. Apolar–apolar ICs, driven by hydrophobic interactions, usually result in weaker (less negative) ΔG\Delta GΔG values than polar or charged interactions. The magnitude of ΔG\Delta GΔG for ICs reflects the strength and nature of their interactions, with stronger binding (more negative ΔG\Delta GΔG) leading to more stable complexes. This is particularly important in applications such as drug design, where optimizing binding affinity is crucial.

The complex formed between Aducanumab and Aβ has already been documented in the Protein Data Bank (PDB ID: 6c03) [33,34]. Leveraging this information, we conducted modeling experiments focusing on Aducanumab, an Alzheimer’s disease treatment recently withdrawn from the market. Our methodology involved pre-processing and standardizing the antibody structure using UCSF Chimera, protein structure prediction using the AlphaFold2, followed by docking analysis using HADDOCK, both before and after linkage with transferrin via the (G4S)n linker. Subsequently, we evaluated binding affinity and interaction patterns through the PRODIGY server, paying particular attention to the linker’s length. The binding parameters calculated included the following (Figure 1).

The retention of antibodies within the brain can be further influenced by receptor recycling pathways, such as those involving the neonatal Fc receptor (FcRn). Such mechanisms are more prominent in peripheral circulation, where FcRn is critical to antibody longevity. Still, their presence in the CNS is limited, and their effect on half-life is less pronounced [35].

The exocytosis of antibodies can be reduced or prevented by linking the transcytosis agent with a cleavable linker that would break when the conjugate enters the brain [36].

Antibody engineering may also involve removing the Fc region; however, this approach has several limitations. When the Fc (Fragment crystallizable) region of an antibody is removed, such as in antibody fragments like Fab (antigen-binding fragment) or scFv (single-chain variable fragment), the half-life within the brain can be significantly shortened [37].

## 4. Moving Forward

Developing new antibodies may take decades and billions in cost. To enable the availability of treatments for neurological disorders that require the entry of an antibody into the brain, we recommend modifying the currently approved antibodies such as lecanemab, donanemab, and aducanumab by conjugating them in vitro with either transferrin protein (UniProt P02787) or its N-methyl lobe [38]. It is essential to understand that an approved antibody modified by conjugating with transferrin protein or a fraction of this protein will be considered a new drug by the US FDA; however, since the resulting compound has already been approved, the developers may be able to secure many concessions in testing, such as nonclinical toxicology. The justification for the conjugation should first be established in animal models. Likely, the clinical dose requirement will be reduced substantially, allowing the FDA to give more favorable consideration to these filings, perhaps in a fast track.

Testing of whether an antibody crosses the blood–brain barrier (BBB) can be performed in animal models commonly used in preclinical studies. However, the degree to which these models mimic human BBB properties varies, which has important implications for the translation of findings.

In vitro methods to study transcytosis in the brain rely on models that replicate the blood–brain barrier (BBB) and assess how molecules traverse it through mechanisms such as receptor-mediated transcytosis (RMT), adsorptive-mediated transcytosis (AMT), or passive diffusion. Among these approaches, Transwell assays are widely used for their simplicity and cost-effectiveness. These assays employ a two-compartment system separated by a membrane seeded with brain endothelial cells, such as hCMEC/D3 or primary human brain microvascular endothelial cells, to mimic the blood and brain sides of the barrier. Molecules introduced into the upper compartment are monitored for transport into the lower compartment, although the system lacks the complex dynamics and cellular interactions of in vivo conditions [39].

Besides static models, microfluidic BBB-on-a-chip systems replicate the BBB’s dynamic environment by incorporating brain endothelial cells, astrocytes, and pericyte co-cultures. These devices simulate fluid shear stress and more closely mimic in vivo conditions, albeit at higher costs and requiring specialized equipment [40]. Similarly, cell culture models using co-cultures or tri-cultures of endothelial cells with astrocytes, pericytes, and neurons provide enhanced mimicry of the neurovascular unit, making them valuable for mechanistic studies. However, scalability remains limited [41].

Fluorescent or radiolabeled tracers are frequently employed to quantify transcytosis, with labeled molecules being tracked across the BBB models using techniques such as fluorescence imaging or autoradiography. However, labeling molecules can alter their intrinsic properties, introducing a limitation to this method [42]. For receptor-mediated pathways, assays like ELISA, surface plasmon resonance (SPR), or flow cytometry help characterize binding affinity and uptake, though these methods do not directly measure transcytosis [43]. Advanced imaging techniques, such as confocal or electron microscopy, provide insights into endocytosis and intracellular trafficking, revealing whether molecules are transported across endothelial cells or degraded within them [44].

Biomimetic membranes coated with BBB-specific proteins offer a reproducible and cost-effective alternative to studying molecular interactions, although they oversimplify the barrier’s structure [45]. The choice of method often depends on study objectives, with Transwell assays and artificial membranes being ideal for initial screenings. At the same time, BBB-on-a-chip systems and co-culture models excel in mechanistic investigations. These in vitro studies are complemented by in vivo rodent models to evaluate BBB permeability and transcytosis further.

In vivo methods allow for a transition from in vitro findings to understanding BBB dynamics in a living system. Techniques such as positron emission tomography (PET) and single-photon emission computed tomography (SPECT) utilize radiolabeled molecules to visualize and quantify brain uptake non-invasively. [46]. SPECT offers a cost-effective alternative, using gamma-emitting radiotracers like Tc-99m, but it provides lower spatial resolution than PET [47]. Magnetic resonance imaging (MRI) and its variant, dynamic contrast-enhanced MRI (DCE-MRI), employ contrast agents such as gadolinium to assess vascular leakage and transcytosis with excellent spatial resolution. However, the sensitivity is lower than that of nuclear imaging methods [48].

Fluorescence molecular tomography (FMT) and near-infrared spectroscopy (NIRS) are non-ionizing alternatives for tracking fluorescently labeled molecules. At the same time, optical imaging through intracranial windows allows the real-time visualization of molecular transport at high spatial and temporal resolution, albeit with minimally invasive procedures [49]. For unlabeled products, methods such as magnetic resonance spectroscopy (MRS), which detects chemical signatures, and pharmacokinetic analysis with cerebrospinal fluid (CSF) sampling are utilized to infer brain penetration [50]. Whole-brain extraction and quantification provide precise measurements of brain concentrations, while mass spectrometry imaging (MSI) offers spatial distribution mapping without labeling [44].

Integrating in vitro models with in vivo imaging techniques and post-mortem tissue analysis provides a comprehensive understanding of transcytosis mechanisms. However, challenges remain due to species-specific differences in BBB structure and function, underscoring the need for caution in translating preclinical findings to humans [39].

However, significant differences between animal models and humans can affect the results. Rodents have a more permeable BBB than humans, which can lead to an overestimation of antibody penetration. Additionally, transport receptors’ expression levels and functionality, such as the transferrin receptor or insulin receptor (often used in receptor-mediated transcytosis strategies), can vary significantly between species. While closer to humans regarding BBB characteristics, non-human primates still do not perfectly replicate human physiology and may not allow for fully predicted clinical outcomes [51].

Species-specific differences in antibody pharmacokinetics and immune responses further complicate the translation of results. For example, human-derived antibodies might elicit animal immune reactions, altering their biodistribution and BBB-crossing efficiency. Genetic engineering techniques, such as the development of humanized mice, can mitigate some of these issues by incorporating human genes for BBB transporters or receptors [52].

In summary, while animal models are invaluable for assessing whether an antibody can cross the BBB, the results must be interpreted cautiously due to interspecies differences. Using a combination of rodent and non-human primate models and complementary in vitro BBB models (e.g., human cell-based systems) provides a more comprehensive understanding and increases the likelihood of successful translation to humans.

However, if the goal is to determine whether an approved antibody can be made more effective and safer, this can be readily demonstrated in any of the above in vitro tests conducted side-by-side.

## 5. Conclusions

Current clinical trials of antibodies intended to enter the brain to treat neurological disorders are unethical unless there is sufficient proof of their reaching into the brain. While the FDA must answer the New York Times report with evidence that it has followed the GAO recommendations in qualifying the IRBs, the FDA must also revise clinical trial guidelines requiring proof of sufficient brain bioavailability at the target site, where known, to be eligible for exposing humans to such trials. The developers must adopt antibody engineering to enhance the transcytosis and preventable exocytosis of antibodies targeting the brain tissue. Additionally, such trials should consider the possibility of significant placebo effects, common for these drugs [31]. It is worth suggesting that the clinical effects reported with a fast action that could not be explained by the transit speed, such as within a minute of the drug reaching the brain, may be due to placebo effects since they all end up with highly toxic effects [29].

The regulatory and approval expenses for these drugs are multi-billions of dollars; the total healthcare costs for treating Alzheimer’s disease were estimated at USD 305 billion in 2020, with projections exceeding USD 1 trillion as the population ages. These figures encompass direct medical care and indirect expenses like informal caregiving. However, despite this high cost, the effects are negligible as insufficient quantities of these drugs enter the brain [53].

The FDA must take action to reduce human abuse in these trials and encourage the development of scientifically rational products that are not currently available in the form of antibodies. Federal law prohibits unnecessary human testing through frameworks such as the Common Rule and the Federal Food, Drug, and Cosmetic Act (FDCA), both of which establish stringent requirements to ensure human research is necessary, ethical, and scientifically valid. Similarly, the FDCA, enforced by the FD, regulates clinical trials for drugs, biologics, and medical devices. Under FDA regulations, sponsors must demonstrate the necessity of human trials by showing that preclinical studies (such as in vitro and animal models) provide sufficient justification for moving to human testing [54]. IRBs are tasked with reviewing protocols under 21 CFR 56.111 to ensure that trials do not expose participants to unjustifiable risks or involve unnecessary duplicative testing. Furthermore, the FDA has the authority under 21 CFR 312.42 to impose a clinical hold to stop trials if they are deemed unnecessary, unsafe, or inadequately justified [55]. Ethical standards are reinforced by international guidelines like the Declaration of Helsinki, which emphasize the need for rigorous justification for human trials and the preference for alternative methods when available to minimize human exposure [56]. Violations of these regulations can lead to severe consequences, including criminal and civil penalties, revocation of federal funding, and institutional and individual liability. Together, these laws and ethical guidelines work to protect human subjects, promote scientifically and ethically sound research, and prevent unnecessary human experimentation.

## Figures and Tables

**Table 1:** Antibodies that failed in the treatment of Alzheimer’s and Parkinson’s Disease.

Monoclonal Antibody	Target	Indication	Stages
Aducanumab [10]	Amyloid-β	Alzheimer’s Disease	Initially approved, then withdrawn due to efficacy concerns and safety issues.
Bapineuzumab [15]	Amyloid-β	Alzheimer’s Disease	Phase III trials did not demonstrate significant cognitive improvements; development was discontinued.
Bepranemab [16]	Tau	Alzheimer’s Disease	Phase II trials did not meet primary endpoints; the development status was uncertain.
Crenezumab [17]	Amyloid-β	Alzheimer’s Disease	Phase III trials did not meet primary endpoints; development was discontinued.
Gantenerumab [18],	Amyloid-β	Alzheimer’s Disease	Phase III trials did not meet primary endpoints; development was discontinued.
GSK933776 [19]	Amyloid-β	Alzheimer’s Disease	Phase II trials did not meet primary endpoints; development was discontinued.
Ponezumab [20]	Amyloid-β	Alzheimer’s Disease	Phase II trials showed no significant efficacy; development halted.
Semorinema [21]	Tau	Alzheimer’s Disease	Phase II trials did not meet primary endpoints; the development status was uncertain.
Solanezumab [22]	Amyloid-β	Alzheimer’s Disease	Phase III trials failed to meet primary endpoints; development halted.
Tilavonemab [16]	Tau	Alzheimer’s Disease	Phase II trials did not meet primary endpoints; development status is uncertain.
Cinpanemab [23],	α-Synuclein	Parkinson’s Disease	Phase II SPARK trial terminated due to lack of efficacy.
Prasinezumab	α-Synuclein	Parkinson’s Disease	Phase II trials did not meet primary endpoints; the development status was uncertain.

**Table 2:** The status of approved antibodies to treat Alzheimer’s Disease across various jurisdictions. None approved for Parkinson’s Disease.

Antibody	United States	European Union	United Kingdom	Japan	China	Australia	Canada
Aducanumab	Approved by the FDA (2021); FDA	Rejected by the EMA (2021); EMA	Not approved	Not approved	Not approved	Not approved	Not approved
Lecanemab	Approved by the FDA (2023); FDA	Rejected by the EMA (2023); Reuters	Approved by the MHRA (2023); not recommended by NICE; Reuters	Approved (2023)	Approved (2023)	Not approved by TGA (2023); Herald Sun	Approved (2023)
Donanemab	Approved by the FDA (2024); FDA	Under EMA review	Approved by the MHRA (2024); not recommended by NICE for NHS; Reuters	Application submitted, pending decision	Application submitted, pending decision	Application submitted, pending decision	Application submitted, pending decision

## Data Availability

Data are contained within the article.
